# Choice of initial antiretroviral drugs and treatment outcomes among HIV-infected patients in sub-Saharan Africa: systematic review and meta-analysis of observational studies

**DOI:** 10.1186/s13643-017-0567-7

**Published:** 2017-08-25

**Authors:** Tadesse Awoke Ayele, Alemayehu Worku, Yigzaw Kebede, Kassahun Alemu, Adetayo Kasim, Ziv Shkedy

**Affiliations:** 1 Department of Epidemiology and Biostatistics, Institute of Public Health, University of Gondar, Gondar, Ethiopia; 2 School of Public Health, Addis Ababa University, Addis Ababa, Ethiopia; 3 Wolfson Research Institute, Durham University, Durham, UK; 4 I-BioStat, Hasselt University, Diepenbeek, Belgium

**Keywords:** Treatment failure, NNRTI switch, First line ART regimen, Systematic review, Sub-Saharan Africa

## Abstract

**Background:**

The effectiveness of antiretroviral therapy (ART) depends on the choice of regimens during initiation. Most evidences from developed countries indicated that there is difference between efavirenz (EFV) and nevirapine (NVP). However, the evidences are limited in resource poor countries particularly in Africa. Thus, this systematic review and meta-analysis was carried out to summarize reported long-term treatment outcomes among people on first line therapy in sub-Saharan Africa.

**Methods:**

Observational studies that reported odds ratio, relative risk, hazard ratio, or standardized incidence ratio to compare risk of treatment failure among HIV/AIDS patients who initiated ART with EFV versus NVP were systematically searched. Searches were conducted using the MEDLINE database within PubMed, Google Scholar, HINARI, and Research Gates between 2007 and 2016. Information was extracted using standardized form. Pooled risk ratios (RR) and 95% confidence intervals (CI) were calculated using random-effect, generic inverse variance method.

**Result:**

A total of 6394 articles were identified, of which, 29 were eligible for review and abstraction in sub-Saharan Africa. Seventeen articles were used for the meta-analysis. Of a total of 121,092 independent study participants, 76,719 (63.36%) were females. Of these, 40,480 (33.43%) initiated with NVP containing regimen. Two studies did not report the median CD4 cell counts at initiation. Patients who have low CD4 cell counts initiated with EFV containing regimen. The pooled effect size indicated that treatment failure was reduced by 15%, 0.85 (95%CI: 0.75–0.98), and non-nucleoside reverse transcriptase inhibitor (NNRTI) switch was reduced by 43%, 0.57 (95%CI: 0.37–0.89).

**Conclusion:**

The risk of treatment failure and NNRTI switch were lower in patients who initiated with EFV than NVP-containing regimen. The review suggests that initiation of patients with EFV-containing regimen will reduce treatment failure and NNRTI switch.

**Electronic supplementary material:**

The online version of this article (doi:10.1186/s13643-017-0567-7) contains supplementary material, which is available to authorized users.

## Background

During the last three decades, HIV/AIDS has become the threat to the world. Almost 75 million people have been infected, and about 36 million people have died of HIV [1]. The introduction of antiretroviral therapy (ART) changes HIV/AIDS from diseases with a high mortality rate to manageable chronic diseases by decreasing the progression of AIDS and reducing HIV-related illness and deaths. Researches revealed that improved access to ART is helping to drive a decline in HIV-related morbidity and mortality [2–5]. In the USA and Canada, a person in his or her 20s who contracts HIV can now expect to live into the 70s if initiated ART early [6].

The standard therapy consists of two nucleoside reverse transcriptase inhibitors (NRTIs) and one non-nucleoside reverse transcriptase inhibitor (NNRTI) [7]. In 2010, these guidelines were revised and recommended less toxic drugs in first-line therapy by replacing stavudine (d4T) with tenofovir (TDF) [8]. In resource-limited countries, World Health Organization (WHO) recommends the use of two NNRTI (nevirapine (NVP) and efavirenz (EFV)) as first-line ART regimen. EFV, combined with two NRTIs, is the recommended option for initial therapy and is the most widely used NNRTI [9].

Staying on an initial regimen medication that successfully suppresses viral replication is essential as it slows disease progression and preserves options for future treatment [3]. However, patients modify or switch their regimen due to different reasons. Toxicity is the most frequently reported reason for modifying or switching the first combined antiretroviral therapy regimens [10]. Once a drug combination is modified, it can no longer be given to the same patient again. It also causes significant morbidity and poor quality of life and also can be an important barrier to adherence, ultimately resulting in treatment failure and viral resistance [11]. Treatment failure due to different reasons is the challenge faced by the current ART scale up program especially in resource-limited countries [12, 13]. Where as the resource-rich countries have documented the effectiveness of the choice of initial regimen [14].

In resource-limited countries, the available evidences are not consistent with the effectiveness of NNRTI choice. In Cameroon, hematologic related adverse drug reaction was high among those who started ART which leads to treatment modification [15]. According to a Ghanaian study, the effectiveness of first-line ART (i.e., the proportion of patients who stay on the initial regimen) was 83.3% depending on virologic failure [16]. Documented virologic failure suggests that access to viral load measurements may actually reduce the rate of switching to a second-line regimen [17]. The substitution due to toxicity of NVP was higher, and according to [18], 8 and 2% substitute their initial regimen when initiated with NVP and EFV, respectively. Study in southern Ethiopia [19] showed that most modifications had occurred during the first 6 months of treatment.

Studies in resource-rich settings revealed that EFV-containing regimen has better treatment outcomes than NVP-containing regimen [20, 21]. In India, a randomized clinical trial [22] also showed that regimen containing NVP was inferior and was associated with more frequent virologic failure and death. Similarly, this pattern was reported in Swaziland, Zambia, and Botswana [23, 24]. However, studies in Ghana and Ethiopia indicated that there is comparable effect between EFV and NVP [16, 25].

The choice of treatment combinations for HIV-infected patients to initiate ART depends on cost and efficacy [7, 26]. Identifying the long-term treatment outcomes of these drugs is very decisive for clinical decision. Clinical decision-making requires ongoing reconciliation of studies that provide different answers to the same question. The above example indicates contradicting results in terms of the effectiveness of the drugs. Though studies showed significantly different effect on long-term treatment outcome in resource-rich settings among NNRTI groups, there was no strong evidence in resource-poor countries. Thus, local evidences as per the real setting of the population will assist the clinicians to focus on the most effective treatment combinations in resource-poor settings. This review aimed to investigate if treatment failure and NNRTI substitution are different between NVP and EFV containing initial regimen.

## Methods

### Search strategies

Comprehensive and exhaustive search strategy was made by two of the investigators to identify all relevant studies. MEDLINE through PubMed, Google Scholar, HINARI, and Research Gates were used to search for the relevant papers. Population, Intervention, Comparison and Outcome (PICO) format was used to search the relevant studies. PROSPERO registration was not done.

For HIV-infected adults a combination of NRTI and NNRTI drugs has been given as first-line regimen. NVP and EFV are used as first-line drugs for most of the patients. The research question was “Does the choice of NNRTI drug affect the effectiveness of first line treatment?” The search strategy included Medical Subject Heading (MeSH) terms and a range of relevant keywords. Combinations of keywords: (((((((((((((HIV) OR AIDS*) AND antiretroviral*) OR HAART*) OR ART*) OR ARV*) AND NNRTI*) AND outcomes*) OR treatment failure) OR switch) OR substitution) OR Discontinuation) AND Africa) OR sub-Saharan Africa. The authors were contacted and requested full articles by email when the article was not accessed from these sources.

### Inclusion criteria

The study eligibility was determined using the following criteria:

*Type of studies*: Epidemiological study designs done in sub-Saharan Africa, including cohort, case-control, and retrospective follow-up, comparative cohort, and analytical cross-sectional studies, were included.

*Intervention*: This review include studies that evaluated EFV compared to NVP-containing regimens in a combination of three antiretroviral drugs. If cohorts report on other drugs in combination with EFV or NVP, or two NRTIs and a protease inhibitor, then only data for combination ART of two NRTIs with NVP or EFV were extracted.

*Types of outcome measures*: This review considered studies that included treatment failure or NNRTI switch as an outcome measure. Studies published between 2007 and 2016 in English language were included.

### Exclusion criteria

Studies which were conducted among children (age < 15 years), published other than English language, and initiated ART other than NNRTI drugs were excluded from the review.

### Study selection

The selection of studies from electronic databases was conducted in two stages: First decision was made based on titles and, where available, abstracts. Second, for studies that met the inclusion criteria, or in cases when a definite decision could not be made based on the title and/or abstract alone, the full paper was obtained for detailed assessment against the inclusion criteria. Two independent reviewers assessed study quality. The Kappa statistics was 0.86 which indicates the presence of good agreement between the reviewers. The papers were given to third reviewer for consensus while a discrepancy in decision process.

### Quality assessment tools

Quality assessment of the included studies was also independently performed using the Joanna Briggs Institute Meta-Analysis of Statistics Assessment and Review Instrument (JBI-MAStARI) [27] and Newcastle-Ottawa quality assessment scale [28] by two independent reviewers. The first assessment tool consisted of nine questions. The later consisted of eight multiple-choice questions that addressed subject selection and comparability (of cases and controls in case-control studies, of cohorts in cohort studies) and the assessment of the outcome (in case-control studies) or exposure (in cohort studies). The number of possible answers per question ranged from two to five. High-quality responses earned a star, totaling up to nine stars.

### Data extraction process

A standardized data collection form [29] was used to extract necessary data from the articles: the title of the study, first author’s last name, country where the study was conducted, study design, year of recruitment and follow-up, year of publication, sample size, study population, diagnosis and identification of treatment modification, average duration of follow-up (for cohort study), potential confounders that were adjusted for, main findings and quality assessment tools. Any data discrepancy was resolved by referring back to the original study.

### Pretesting the data extraction tool

The selection process and data collection tool was pretested based on the inclusion criteria on five articles. It was aimed to check reliability of interpretation and classification of the studies appropriately and to ensure that all the relevant information was captured. The consistency of extracted data was assessed to reduce data extraction errors.

### Outcome measures

Treatment failure was defined as either virologic, clinical, or immunological failure as per the definition of WHO ART guideline [8]. Studies which used composite outcome as their event was also defined as treatment failure. NNRTI substitution was defined as either NNRTI modification, regimen change, NNRTI resistance, or NNRTI discontinuation.

### Data synthesis and statistical analysis

Heterogeneity among studies was examined using *I*-squared statistic. According to the test, *I*-square estimate greater than 50% was considered as indicative of moderate to high levels of heterogeneity [30]. Adjusted point estimates were extracted from individual studies and combined together to calculate the pooled estimates. The DerSimonian-Laird random effects method was used to incorporate an additional between study component to the estimate of variability [31, 32]. Subgroup analyses were done to explore differences in outcomes according to study outcomes. The qualitative and quantitative methods were used to present the data extracted from each study. Funnel plot and Egger’s test were used to check the presence of publication bias [33]. We plotted the effects by the inverse of its standard error. The symmetry of such plots was assessed both by using visually and with Egger’s test to see if the effect decreased with increasing sample size. Since graphical evaluation can be subjective, we conducted a regression asymmetry test as formal statistical tests for the presence of publication bias.

Meta-regression was conducted to investigate the impact of study characteristics on the study estimates of relative risk. The natural logarithm of the risk ratio was the dependent variable, and length of follow-up, median baseline CD4 cell counts, median age, proportion of female and year of publication were entered as explanatory variable. *P* value and 95% confidence interval were used to test statistical significant. Statistical analysis was performed using Stata version 12 software and Review Manager Version 5.3. Stata does not have built-in meta-analysis command; however, user written command called *metan* is available. Steps to install the command can be obtained from the book of Egger [34]. PRISMA 2009 checklist was used to keep the standard of the report (see Additional file 1).

## Results

**Fig. 1:**
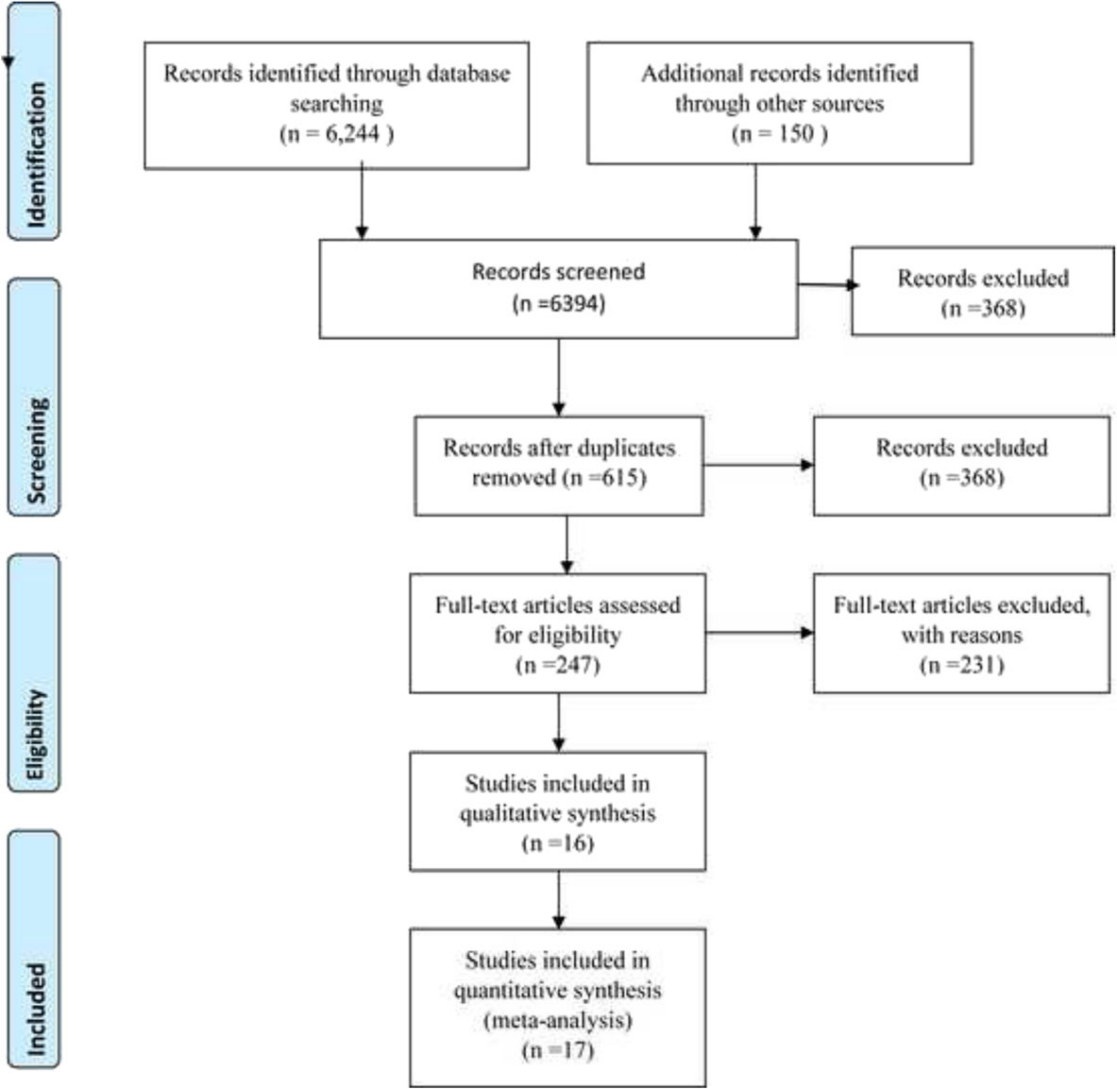
The PRISMA flow diagram of identification and selection of studies for the systematic review and meta-analysis

For inclusion in this review, studies were required to provide comparisons of NVP and EFV on the risk of long-term outcomes. A total of 6394 articles were identified in English language and human domain restrictions, of which, 5779 were rejected by looking only at the title of the research. The remaining 615 articles were further screened, and subsequently, 395 were considered irrelevant or duplicates. The abstracts of 238 articles were then evaluated independently. Of these, 158 records were excluded because of no comparison groups of the outcomes of interest, missing comparison of EFV versus NVP drugs and reviews and meta-analysis. The PRISMA flow diagram [35] is used to present stages of review process (Fig. 1).

A study done on comparison between NVP and lopinavir-ritonavir [36] was excluded as it was not the interest of this review. Other six papers were excluded as the studies were conducted among children [37], conducted outside of sub-Saharan Africa, [38, 39, 40, 41], and systematic reviews and meta-analysis articles [20] were. Subsequently, of 79 full record articles, a total of 36 were eligible studies. Further, the full text of 36 articles were reviewed in detail, and 20 of them were excluded due to lack of sufficient information on sample size, design, and analysis. Study [42] used case-control design, and the sample size was small for both the cases and controls. It was not also clear how the size was determined. Study [43] had used cross-sectional study design, and the assessment tools might not evaluate the quality appropriately. Therefore, 16 studies were included in the quantitative synthesis out of which 17 outcome measures were identified for meta-analysis.

### Quality assessment

Two independent reviewers assessed the articles prior to inclusion to maintain methodological validity using the Joanna Briggs Institute Meta-Analysis of Statistics Assessment and Review Instrument (JBI-MAStARI) [27]. The scores ranged from 5/9 to 8/9 in absolute number and 55.6 to 88.9% in percentage. In addition, Newcastle-Ottawa quality assessment scale was used. The scores for each study ranged from 4 to 7 stars (from a total of 9).

### Characteristics of included studies

All the 16 studies were conducted between 2007 and 2016. Sample size ranges from 167 [43] to 27,350 [44] patients. A total of 70,537 patients were included in all the studies. Of whom, 45,010 (63.8%) were females. The proportion of females ranges from 51 to 72%. Most of the patients, 42,039 (59.6%), were initiated with EFV-containing regimen. Overall, more females were initiated with NVP-containing regimens. The median follow-up time was 4 years (IQR 3–7). Study [45] had the longest follow-up time whereas studies [46, 47] had followed for shorter periods.

Almost half of the studies were from South Africa [43–49], the rest were from Kenya [50, 51], Ghana [10, 52], Nigeria [42], Zambia [50], Ethiopia [25, 53], and sub-Saharan Africa [54, 55]. Study [50] was a multicenter study (in Kenya, Zambia, and Thailand), and data from Kenya and Zambia were taken due to inclusion criteria. A total of 509 and 152 patients were included in Zambia and Kenya, respectively. With regard to the study design, most were retrospective cohort [9]. The minimum and maximum median age for the included studies were 32 (IQR 28–36) and 40 (IQR 35–47) years, respectively. In almost all studies, high median age corresponds to EFV-containing regimen at initiation. The median CD4 cell counts ranges from 67 (IQR 21–161) to 192 (IQR 112–324). The median CD4 cell count was smaller for patients who initiated with EFV-containing regimen. This might be due to the occurrence of different opportunistic infection among this group of patients, and EFV-containing regimen had no organ damage like hepatotoxicity and preferred for this group at large to maintain adherence [8]. Two studies [25, 43] did not report the median CD4 cell count at initiation. Only two studies [45, 55] reported the log transformed median viral load. NRTI backbones used differed between studies. Stavudine (d4T)/3TC were used in 13 studies, and three studies did not use this NRTI backbone at all. AZT/3TC was used in 14 studies, and two studies did not use this backbone at all. TDF/3TC was used less frequently, in only seven studies.

Most (11/16) of the studies used Cox proportional hazards model for the analysis and reported adjusted hazard ratio. Another two studies used stratified and random effect Cox-proportional models. About seven studies used second model (Conditional logistic regression, Poisson regression, mixed effect model and marginal structural models). Two of the studies further used sensitivity analysis. In general, with the statistical model used, most of the articles utilized appropriate analysis methods (Table 1).

**Table 1:** Baseline characteristics of patients on NVP and EFV by study design

Author	Country	Design	Sample size	Follow-up Period	Female	Age Median ± IQR	CD4 count Median ± IQR	Viral load (log) Median ± IQR	BMI Median ± IQR
Stringer et al. [50]	Zambia and Kenya	Prospective Cohort	509 152	4 years	661	Zambia 32 (28–36) Kenya 32 (28–36)	148 (88–211) 147 (76.5–212.5)	5.0 (4.4–5.4) 5.3 (4.8–5.9)	19.7 (18.3–21.9) 19.9 (17.7–22.6)
Kwobah et al. [51]	Kenya	Case-control	3233	5 years	1992	Case: 36.3(30.6–43.2) Control: 36.5(30.7–43.1)	Case: 80 (32–177) Control: 194 (112–324)	NA	NA
Nachega et al. [45]	South Africa	Cohort	1817	10 years	1771	NVP: 33.2 (7.68) EFV: 36(8.0)	NVP: 171(78–243) EFV: 136(58–216)	NVP: 5.1(4.6–5.6) EFV: 5.2(4.7–5.7)	NA
Boulle et al. (18, 48)	South Africa	Cohort	2679	4 years	1896	32(28–38)	83(34–140)	5.5(4.6–5.5)	NA
Shearer et al. ([47])	South Africa	Cohort	12,840	8 years	7962	EFV: 37.2(31.9–43.9) NVP: 33.0(28.4–39.1)	EFV: 98(36–169) NVP: 100.5(42–160)	NA	EFV: 21.7 (19.2_24.9) NVP: 22.0 (19.7_25.1)
Sarfo et al. [52]	Ghana	Retrospective Observational	3990	7 years	2717	EFV: 40 (35–47) NVP: 35 (30–41)	EFV: 127 (45–213) NVP: 140 (56–220)	NA	EFV: 19.6 (17.4–22.3) NVP: 19.9 (17.6–22.9)
Shearer et al. [46]	South Africa	Cohort	2385	1 year	1485	EFV: 37.8 (31.8–44.3) NVP: 33.1 (28.1–40.4)	EFV: 132 (58–194) NVP: 132 (66–179)	NA	EFV: 22.5 (19.7–25.9) NVP: 23.0 (20.2–25.6)
Barth et al. [49]	South Africa	Retrospective observational cohort	735	2–4 years	526	Mean = 35.3	68 (20–140)	Mean = 4.9	NA
Gsponer et al. [54]	Sub-Saharan Africa	Collaborative analysis	2404	6 years	1276	Switched: 29 (25–33) Not switched: 29 (25–34)	Switched: 77 (41–133) Not switched: 146 (82–232)	NA	NA
Sarfo et al. [10]	Ghana	Retrospective cohort study	3999	7 years	2727	38 (32–45)	132 (50–217)	NA	19.8(17.5–22.7)
Keiser et al. [55]	Sub-Saharan Africa	Case control	4281	5 years	3022	Switch: 35 (30–41) Not switch: 34 (30–40)	Switch: 73 (23–133) Not switch: 67 (21–161)	Switch: 5.0 (4.4–5.6) Not switch: 5.3 (4.6–5.8)	NA
Anlay et al. [53]	Ethiopia	Retrospective follow-up	410	5 years	265	33.3 ± 8.7	162.5 (90.5–235.5)	NA	NA
van Zyl et al. [43]	South Africa	Retrospective	167	4 years	NA	NA	NA	NA	NA
Abah et al. [42]	Nigeria	Cohort	6309	3 years	4156	35 (30–42)	154 (76–259)	NA	NA
Bock et al. [44]	South Africa	Multicentre cohort	27,350	4 years	16,828	EFV: 35.8 (30.7–42.6) NVP: 31.1 (27.1–35.8)	EFV: 107 (51–160) NVP: 132 (76–177)	NA	NA
Tirfe et al. [25]	Ethiopia	Retrospective cohort study	492	3 years	289	EFV: 35(10) NVP: 33(11)	NA	NA	NA

NA=Not Applicable

### Treatment failure

In this review, treatment failure, the primary outcome of interest, was measured using clinical, virological, and immunological criteria. Studies [10, 25, 45–47, 49–51, 54, 55] defined treatment failure as their primary outcomes. A total of 30,069 patients were included in the ten studies. Of which, 19,584 (65%) were females. The majority, 17,950 (60%), were initiated with EFV-containing regimen. A total of 4842 patients experience treatment failure for both EFV and NVP drugs (2077 EFV and 2765 NVP). Study [45] defined treatment failure using two separate (consecutive or non-consecutive) measurements of viral load ≥ 400 copies/ml, or switch to another NNRTI or protease inhibitor after at least one such measurement. About 1822 (64.7%) patients were started on EFV-containing regimen. The two groups did not differ in viral load measurement; however, patients started on EFV had a significantly shorter time to virologic suppression. Subsequently, patients started on NVP were more likely to experience virologic failure (20.4 vs 13.8%). For study [50], the outcome was assessed at 48 weeks after initiating ART. Participant was considered as having failed at 48 week if she died prior to that time, or had a plasma viral load ≥ 400 copies/ml (confirmed with repeat testing) at either the 24- or 48-week study visits. The difference in failure rates between the NVP-exposed and unexposed groups was 6.9%. Study [51] was a case-control study in which case defined as adult at least one viral load measurement > 5000 copies/ml or meet the WHO 2006 immunological or clinical failure criteria [56]. Controls were those on non-failing first-line ART with a CD4 count > 400/ml within the last 12 months, at the time of case incidence. Patients who were either pregnant or co-infected with tuberculosis at the time of ART initiation were excluded. A total of 1084 cases were included with median time to ART failure of 37 months. Study [10] defined the outcome measure of treatment failure was a composite of death, clinical progression or discontinuation of NNRTI for any reason. A total of 3999 patients were involved from whom 2369 (59%) initiated by EFV-containing ART and 633 (26.7%) experienced at least one event.

The second outcome of interest was NNRTI substitution. Studies [10, 42, 44, 48, 53] defined NNRTI skin rash, NNRTI discontinuation, regimen change, NNRTI substitution, and regimen change as the outcome measure, respectively. Study [25] defined immunologic response, and study [49] patients retention as the outcome measure. In all studies, the initial NNRTI drug was substituted by another drug in the same regimen, hence defined NNRTI substitution as the outcome measure. Studies [10, 44, 46, 47] had outcome measure of death as an event. There were high number of death in patients who initiated with EFV, 208 (8.8%), than NVP, 110 (6.8%), containing regimen in study [10]. A total of 27,350 patients were included of whom 19,441 (71.1%) started EFV and 7909 (28.9%) started NVP treatment. At the end of the study period, 1593 (5.8%) patients died. Study [47] included 12,840 patients of whom 1061 died (8.3%) within the first 12 months on ART (Table 2).

**Table 2:** Selected results of the included studies

Author	Outcomes	Intervention	Confounder adjusted	Main findings	Newcastle-Ottawa scale
Stringer et al. [50]	Treatment failure	*n*_NVP_ = 355 *n*_EFV_ = 523	Month on ART, CD4 cell count, viral load, WHO stage, age, Hgb, BMI, Weight, NNRTI, TB at baseline	-Month on ART, CD4 cell count, viral load, WHO stage, age, Hgb, and BMI were significantly associated factors. -In total, 724 women (82%) completed 48 weeks of follow-up on an NNRTI-containing regimen	Selection 2 stars Comparability 1 star Outcome 2 stars
Kwobah et al. [51]	Treatment failure	*n*_EFV_ = 427 *n*_NVP_ = 2633	Education level, CD4 category, WHO stage, BMI, Hemoglobin, Adherence, disclosure, travel time, NNRTI, and NRTI	-No association between the choice of NNRTI used (Nevirapine or Efavirenz) and treatment failure -Low baseline CD4, AZT based NRTI, imperfect adherence are associated with first line ART failure	Selection 3 stars Comparability 2 stars Outcome 1 star
Nachega et al. [45]	Virologic failure	*n*_EFV_ = 212 *n*_NVP_ = 103	Age, sex, race, baseline CD4, baseline VL, NRTI, year of ART, adherence	-Nevirapine was associated with greater risk of virologic failure compared to efavirenz -NNRTI, age, sex, baseline viral load, year on ART, and adherence were significantly associated with failure	Selection 2 stars Comparability 2 stars Outcome 2 stars
Boulle et al. [18, 48]	NNRTI substitution	*n*_EFV_ = 1612 *n*_NVP_ = 1067	Weight, age, WHO stage per increment, CD4 Count, district	-Substantial difference in the tolerability of commonly used first line ART drugs. -Baseline weight, and Age, for NVP and Weigh and WHO stage for EFV	Selection 2 stars Comparability 2 stars Outcome 2 stars
Shearer et al. [47]	Treatment failure	*n*_EFV_ = 11,962 *n*_NVP_ = 878	NNRTI, ART year, sex, age, baseline CD4, WHO stage, BMI, NRTI, baseline anemia	-Patients with NVP-are more likely to experience virologic failure -NNRTI, years of ART initiation, age, and baseline CD4 cell counts	Selection 3 stars Comparability 2 stars Outcome 1 star
Sarfo et al. [10]	Composite endpoint	*n*_EFV_ = 2369 *n*_NVP_ = 1621	Sex, age, NNRTI, NRTI, Baseline CD4, baseline BMI, WHO stage, adherence	-Treatment outcomes were comparable whether EFV or NVP is used -There was a 36% lower risk of all-cause discontinuation of EFV compared with NVP -NRTI, age, baseline CD4 counts, BMI, WHO stage, and adherence were associated factors of treatment failure	Selection 3 stars Comparability 2 stars Outcome 2 stars
Shearer et al. [46]	Treatment failure	*n*_EFV_ = 2254 *n*_NVP_ = 131	NNRTI, sex, age, baseline CD4, WHO stage, Anemia, BMI	-Given TDF as NRTI, Nevirapine has higher risk of treatment failure as compared to EFV. -Regimen, and Baseline CD4 cell counts were significantly associated with failure	Selection 3 star Comparability 1 star Outcome 1 star
Barth et al. [49]	Treatment failure	*n*_EFV_ = 426 *n*_NVP_ = 309	Gender, age, BMI, Karnofsky score, CD4 counts, time since start ART, NNRTI, Occupation	-No difference between EFV and NVP in treatment failure −60% of patients showed virological failure; only few of them were switched to second-line treatment -Gender, mean BMI, and baseline CD4 counts were associated in the univariate	Selection 2 star Comparability 1 star Outcome 2 star
Gsponer et al. 2012 [54]	Treatment failure	*n*_EFV_ = 186 *n*_NVP_ = 2218	Age, Sex, baseline CD4, WHO stage	-Mortality was lower among patients who switched compared to patients remaining on failing first-line ART -Current CD4 count was associated	Selection 2 star Comparability 1 star Outcome 1 star
Sarfo et al. [10]	NNRTI Substitution	*n*_EFV_ = 2378 *n*_NVP_ = 1621	NNRTI, gender, age, BMI, WHO stage, CD4 counts, hepatitis B surface antigen status, ALT	-Patients starting nevirapine are more likely to develop rashes and then more likely to discontinue therapy than those starting efavirenz. -NNRTI, gender, and low baseline CD4 counts were associated factors	Selection 3 star Comparability 2 star Outcome 1 star
Keiser et al. [55]	Treatment failure	*n*_EFV_ = 1951 *n*_NVP_ = 2325	Not controlled	-Compared to patients who remained on non-failing first-line therapy, mortality and loss from follow-up was higher in patients who switched, and substantially higher in patients who remained on failing first-line therapy	Selection 2 star Comparability 1 star Outcome 1 star
Anlay et al. [53]	NNRTI Substitution	*n*_EFV_ = 289 *n*_NVP_ = 121	Weight, WHO stage, TB on initial regimen, NRTI, NNRTI, Co-medication, and side effect	-There is no difference in regimen change between NVP and EFV -WHO stage, TB status, co-medication, and side effect were associated factors	Selection 3 star Comparability 2 star Outcome 2 star
van Zyl et al. [43]	NNRTI resistance	*n*_EFV_ = 82 *n*_NVP_ **=** 85	Age, gender, ART regimen, most recent CD4 count, concurrent viral load, and genotypic resistance information	-Failure on NVP therapy may result in cross-resistance to ETV. NNRTI and estimated period of failure were associated	Selection 2star Comparability 2 star Outcome 1 star
Abah et al. [42]	NNRT substitution	*n*_EFV_ = 558 *n*_NVP_ **=** 5751	Sex, age, HBV, CD4 count, WHO stage, NNRTI, NRTI	-Drug substitutions of efavirenz (EFV) were more likely than of nevirapine (NVP) -Age, greater immunosuppression, EFV compared to NVP, and drug toxicity were significant predictors	Selection 2 star Comparability 1 star Outcome 2 star
Bock et al. [44]	NNRT substitution	*n*_EFV_ **=** 19,441 *n*_NVP_ **=** 7909	NNRTI, NRTI (AZT & D4T), gender, age, baseline CD4, WHO stage, TB treatment at baseline, year of ART initiation, level of care	-Superior efficacy of EFV compared with NVP for first-line ART -NNRTI, gender, year of initiation, and province were associated factors	Selection 3 stars Comparability 2 stars Outcome 2 stars
Tirfe et al. [25]	Treatment failure	*n*_EFV_ = 246 *n*_NVP_ = 246	NNRTI, facility type, age, sex, marital status, education status, religion, NRTI, presence of OIs, eligibility criteria, WHO stage, functional status, BMI, provision of IPT, and baseline CD4 counts	-NVP and EFV based HAART regimens were effective and comparable, in term of immunological responses. -Gender, eligibility criteria, WHO stage, provision of IPT, and baseline CD4 counts were associated factors	Selection 3 stars Comparability 2 stars Outcome 2 stars

### Meta-analysis results

**Fig. 2:**
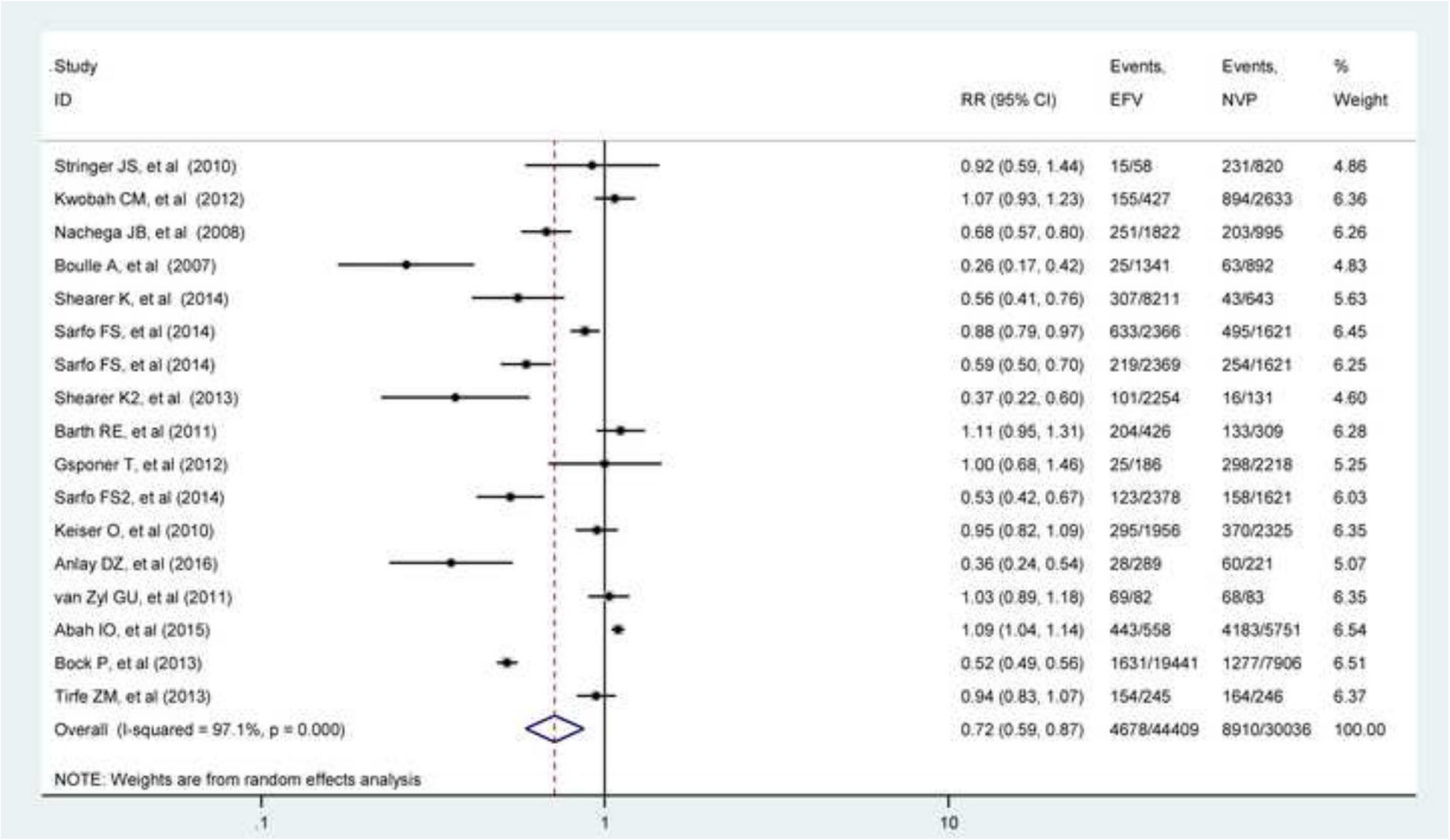
Relative risk of composite outcome associated with the choice of NNRTI drugs regiment during ART initiation

The overall analysis revealed presence of heterogeneity among the individual studies (*I*-squared = 97.1%) (Fig. 2). Subgroup analysis was performed based on the two outcomes of interest mentioned above.

The first subgroup was treatment failure with ten studies [10, 25, 45–47, 50, 51, 54, 55, 57], and the second subgroup was NNRTI substitution which includes studies [10, 42–44, 48, 53]. The forest plot for treatment failure subgroup revealed that studies [25, 49, 51, 54, 55] were not statistically significant, but studies [10, 45–47] showed significant risk of treatment failure individually.

**Fig. 3:**
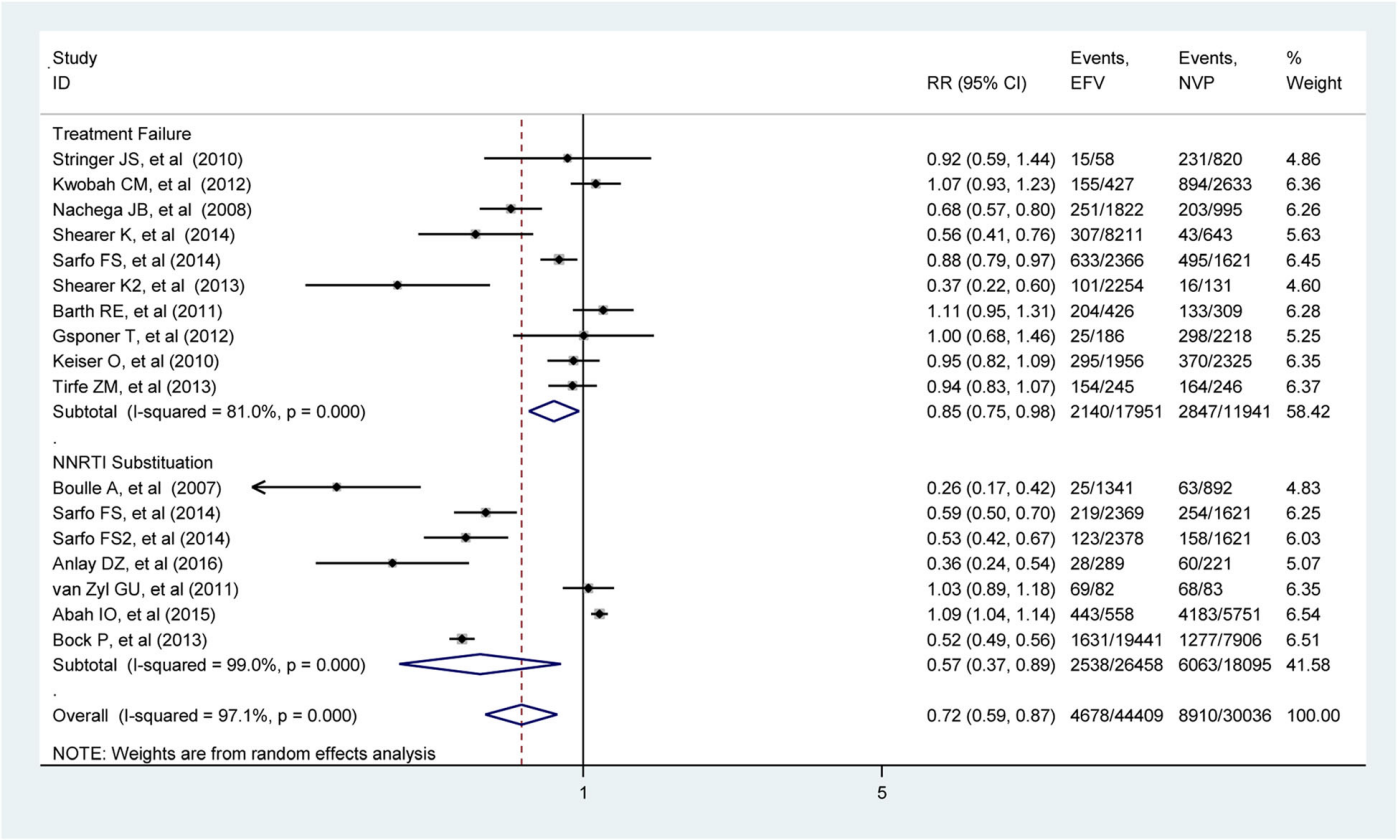
Relative risk of treatment failure and NNRTI substitution associated with the choice of NNRTI drugs regiment during ART initiation

Heterogeneity among the studies within the subgroup was tested using *I*-squared statistics. The *I*-squared value for treatment failure subgroup was found to be 81.0% (*p* value < 0.0001) which indicated the presence of heterogeneity between studies. The weights of the studies were reported from random effect model which ranged from 0.31% to a maximum of 28.28%. The pooled estimate of risk ratio from random effect model was 0.85 (RR = 0.85; 95%CI 0.75–0.88) for EFV than NVP for treatment failure. For NNRTI substitution subgroup, almost all the studies were individually significant except study [43]. The *I*-squared value is 98.9% (*p* value = 0.0001) which indicates as there is high heterogeneity between studies. The weight of the studies ranges from 0.37 to 38.09%. The pooled estimate from random effect model was 0.57 (RR = 0.57; 95% CI 0.37–0.89) which is consistent with the estimate from the fixed effect model (Fig. 3).

### Evaluation for publication bias

One of the main problems in systematic review and meta-analysis is that not all studies carried out are published. Those which are published may be different from those which are not. Research with statistically significant results is more likely to be submitted and published than work with null or non-significant results. This could introduce bias during systematic review and meta-analysis. The presence of publication bias was assessed by funnel plots and tested using Egger’s test which is proposed by Egger et al. [33] to test for asymmetry of the funnel plot.

**Fig. 4:**
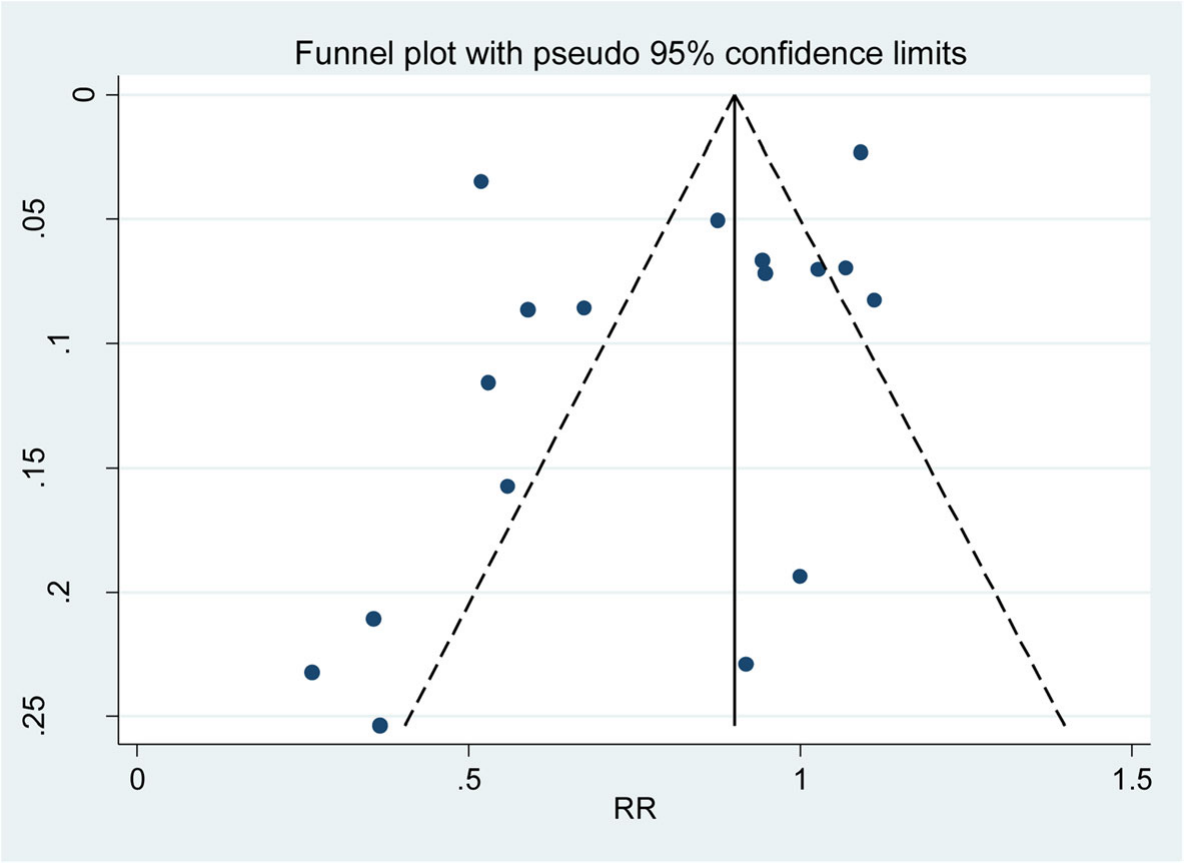
Funnel plot of effect estimates against standard error of log estimate

The funnel plot is assumed to be symmetric in the absence of research bias. The solid vertical line represents the summary estimate of the treatment effect. The diagonal lines representing the 95% confidence limit around the summary treatment effect. These show the expected distribution of studies in the absence of heterogeneity or of selection biases. It seems as there are more studies which lie to the left of the funnel plot. Egger’s test was performed for each subgroup. The test revealed that there was no significant bias for either of the outcome (overall test: intercept = − 2.217, 95% CI − 5.562; 1.128 and *p* value = 0.178) (Fig. 4).

Meta regression was performed to determine whether there is a significant association between independent variables in the form of study versus the dependent variable. A regression model is constructed for covariates, length of follow-up, median CD4 cell counts, median age, and year of publication, and proportion of female. There was no significant relationship between any of the covariates and treatment failure which indicates that these covariates may not be the source of observed variability (Table 3). Bubble plot was plotted for selected covariates (see Additional file 2).

**Table 3:** Parameter estimates of meta-regression

Covariate	Estimate	S.E	95%CI
Length of follow-up	− 0.0138	0.0666	(− 0.1322–0.1046)
Median CD4 count	− 0.0034	0.0066	(− 0.0150–0.0079)
Median age	0.0415	0.0503	(− 0.1013–0.0755)
Year of publication	0.0637	0.0815	(− 0.1736–0.0980)
Female proportion	− 0.5305	1.4575	(− 3.6792–2.6183)

**Fig. 5:**
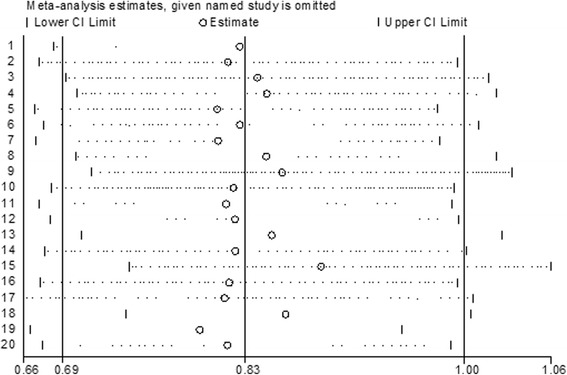
Plot of sensitivity analysis to assessing the influence of individual study

Sensitivity analysis has been performed to identify study which has more influence on the estimates. The plot visually provides estimate with 95% confidence interval, naming the omitted study on the left margin. The lower and upper confidence interval limits were presented for the estimates. The sensitivity analysis revealed that there is no single study affecting the estimate too much. Exclusion of [53] seems influential, but the effect is not statistically significant (Fig. 5).

## Discussion

This systematic review and meta-analysis attempted to assess the individual and pooled estimate of the choice of NNRTI drugs on treatment failure and NNRTI switch in resource-poor settings. A total of 16 observational studies were found which compares EFV versus NVP, out of which, 17 outcome measures were identified in two groups.

The findings revealed that initiation of ART with EFV-containing regimen is associated with a reduced risk of treatment failure (RR = 0.85, 95%CI 0.75–0.98) as compared to NVP-containing regimen in resource-limited settings. This finding was consistent across four of the ten individual studies. This is in line with previous meta-analysis [20] conducted from ten RCTs and 24 observational studies which concluded that EFV-based first line ART regimen is significantly less likely to lead to virologic failure compared to NVP-based ART regimen. This might be due to the hepatotoxic nature of NVP which may lead to poor adherence which might further resulted in treatment failure.

In the 2NN group study [58], there was no any evidence that EFV is superior to NVP twice daily in terms of treatment failure. A Cochrane review of seven randomized clinical trials [59] demonstrated that the two drugs provided comparable levels of viral suppression in patients infected with HIV when combined with two NRTIs. In a non-randomized longitudinal cohort study conducted in India [39], equivalent immunological response was observed among NVP and EFV-based ART.

The risk ratio of NNRTI switch reduced by 0.57 (95% CI 0.37–0.89) times for patients who initiated with EFV than NVP. This finding is consistent with a multicenter randomized non-inferiority trial [60] in which the switching rate is higher among patients who initiated with NVP than EFV. This finding is also consistent with previous meta-analyses [21] which revealed that adults on NVP were two times more likely to discontinue treatment due to any adverse event compared to patients on EFV. Another meta-analysis on five randomized clinical trials and four retrospective clinical trials [61] revealed that the discontinuation rate was high among those who initiated with NVP than EFV which is consistent with this review. Similar finding was reported by meta-analysis of 26 RCTs [62] in which the discontinuation rate was lower among those who initiated with EFV than NVP-containing regimen.

The source of heterogeneity was assessed using meta-regression. Covariates length of follow-up, median CD4 cell counts, median age, and year of publication were included in the regression model. The log relative risk of treatment failure was not a significant difference among length of follow-up, median CD4 cell counts, median age, or year of publication. This might be due to the small number of included studies in the analysis. Sensitivity analysis also revealed that there is no single study which influences the pooled estimate.

These results need to be interpreted with caution due to limitations. Although a lot of efforts have been made to find more studies, still there were few studies which satisfied the inclusion criteria. The analysis was limited to only articles published in English language; the evidence may not be sufficiently robust to determine the comparative effectiveness of EFV and NVP due to the size of included studies. In addition, the analysis included articles with different definitions of treatment failure and different lengths of follow-up. The reviewed articles have also differences in study design, the type of statistical methods, and the variables included in the analysis. These variations may have resulted in selection bias or low statistical power, thus hindering results. Most of our analyses detected heterogeneity between effect estimates obtained across studies. DerSimonian and Laird random effect model was used to determine the pooled effect size [32, 33]. However, the source of variation might not be real heterogeneity rather within study differences which may introduce bias on the pooled effect size.

## Conclusion

The finding of this review showed that initiation of ART with EFV-containing regimen has reduced risk of treatment failure as compared to NVP-containing regimen. In addition, the patients who initiated with EFV are less likely to switch than those with NVP. In contrast, there was about 50% increased risk of death in patients who initiated with EFV as compared to NVP-containing regimens. Even though EFV is more expensive to afford for resource-poor settings, initiating the patient with EFV-containing regimen could be supreme important.

## Additional files

Additional file 1: Preferred Reporting Items for Systematic Reviews and Meta-Analyses (PRISMA) 2009 checklist. The PRISMA 2009 checklist which was used to standardize the review. (PDF 589 kb)

Additional file 2: Bubble plot with fitted meta-regression line depicting the relationship between the risk of composite outcomes and baseline covariates (proportion of female, baseline CD4 cell counts and follow up period). Fig. S1. Bubble plots of the fitted meta-regression with proportion of female, baseline CD4 counts and follow-up time. (PDF 177 kb)

## Abbreviations

AIDS: Acquired immune deficiency syndrome
ART: Antiretroviral therapy
AZT: Zidovudine
CI: Confidence intervals
CNS: Central nervous system
EFV: Efavirenz
GI: Gastrointestinal
HIV: Human immunodeficiency virus
NNRTIs: Non-nucleoside reverse transcriptase inhibitors
NRTIs: Nucleotide reverse transcriptase inhibitors
NVP: Nevirapine
OR: Odds ratio
PIs: Protease inhibitors
PRISMA: Preferred Reporting Items for Systematic Reviews and Meta-Analyses
WHO: World Health Organization
