# Supplementary material for: Choice of initial antiretroviral drugs and treatment outcomes among HIV-infected patients in sub-Saharan Africa: systematic review and meta-analysis of observational studies
Source: Syst Rev. 2017 Aug 25;6:173. doi: 10.1186/s13643-017-0567-7 (PMC5574138; doi:10.1186/s13643-017-0567-7)
Supplement: Supplementary file 1 — Preferred Reporting Items for Systematic Reviews and Meta-Analyses (PRISMA) 2009 checklist. The PRISMA 2009 checklist which was used to standardize the review. (PDF 589 kb) [file 13643_2017_567_MOESM1_ESM.pdf]

PRISMA 2009 Checklist

Section/topic	#	Checklist item	Reported on page #
**TITLE**			
**Choice of Initial Antiretroviral Drugs and Treatment Outcomes among HIV-Infected Patients in sub-Saharan Africa: Systematic Review and Meta-analysis of Observational Studies**	1	Identify the report as a systematic review, meta-analysis, or both.	1
**ABSTRACT**			
Background: Most evidences from developed countries indicated that there is difference between efavirenz (EFV) and NVP nevirapine (NVP). However, the evidences are limited in resource poor countries particularly in Africa. Thus, this systematic review and meta-analysis was carried out to summarize reported long-term treatment outcomes among people on first line therapy in sub-Sharan Africa. **Methods:** Observational studies that compared risk of treatment failure among HIV/AIDS patients initiated ART with EFV versus NVP were systematically searched. Information was extracted using standardized form. Pooled risk ratios (RR) and 95% confidence intervals (CI) were calculated using random-effect, generic inverse variance method. ***Result:*** A total of 5394 articles were identified, of which 29 were eligible for review and abstraction in sub-Sharan Africa. Seventeen articles were used for the meta-analysis. Of a total of 121092 independent study participants, 76719 (63.36%) were females. Of these, 40480 (33.43%) initiated with NVP containing regimen. Two studies did not report the median CD4 cell count at initiation. Patients who have low CD4 cell counts initiated with efavirenz containing regimen. The pooled effect size indicated that treatment failure was reduced by 15%, 0.85 (95%CI:0.75-0.98), and non-nucleoside reverse transcriptase inhibitor (NNRTI) switch was reduced by 43%, 0.57 (95%CI:	2	Provide a structured summary including, as applicable: background; objectives; data sources; study eligibility criteria, participants, and interventions; study appraisal and synthesis methods; results; limitations; conclusions and implications of key findings; systematic review registration number.	2

0.37-0.89). ***Conclusion:*** The risk of treatment failure and NNRTI switch were lower in patients who initiated with EFV than NVP containing regimen. The review suggests that initiation of patients with EFV containing regimen will reduce treatment failure and NNRTI switch.			
**INTRODUCTION**			
The choice of treatment combinations for HIV-infected patients to initiate ART depends on cost and efficacy. Identifying the long-term treatment outcomes of these drugs is very decisive for clinical decision. Clinical decision-making requires ongoing reconciliation of studies that provide different answers to the same question. The above example indicate contradicting results in terms of the effectiveness of the drugs. Though studies showed significantly different effect on long-term treatment outcome in resource rich settings among NNRTIs groups, there was no strong evidence in resource poor countries. Thus, local evidences as per the real setting of the population will assist the clinicians to focus on the most effective treatment combinations in resource poor settings.	3	Describe the rationale for the review in the context of what is already known.	4
This review aimed to investigate if treatment failure and NNRTI substitution are different between NVP and EFV containing initial regimen.	4	Provide an explicit statement of questions being addressed with reference to participants, interventions, comparisons, outcomes, and study design (PICOS).	4
**METHODS**			
No review protocol is used	5	Indicate if a review protocol exists, if and where it can be accessed (e.g., Web address), and, if available, provide registration information including registration number.	
Eligibility criteria<br>**Type of studies**: Epidemiological study designs done in sub-Saharan Africa, including cohort, case-control, retrospective follow up, comparative cohort, and analytical cross-sectional studies were included.	6	Specify study characteristics (e.g., PICOS, length of follow-up) and report characteristics (e.g., years considered, language, publication status) used as criteria for eligibility, giving rationale.	5

**Intervention:** This review included studies that evaluated EFV compared to NVP-containing regimens in a combination of three antiretroviral drugs. If cohorts report on other drugs in combination with EFV or NVP, or two NRTIs and a protease inhibitor, then only data for combination ART of two NRTIs with NVP or EFV were extracted.<br>**Types of outcome measures:** This review considered studies that included treatment failure or NNRTI switch as an outcome measure. Studies published between 2007 and 2016 in English language were included.<br>**Exclusion Criteria**<br>Studies which were conducted among children (age<15 years), published other than English language, and initiated ART other than NNRTI drugs were excluded.			
**Information sources**<br>MEDLINE through PubMed, google scholar, HINARI, and Research Gates were used to search for the relevant papers.	7	Describe all information sources (e.g., databases with dates of coverage, contact with study authors to identify additional studies) in the search and date last searched.	4
**Search**<br>Comprehensive and exhaustive search strategy was made by two of the investigators to identify all relevant studies	8	Present full electronic search strategy for at least one database, including any limits used, such that it could be repeated.	4
**Study selection**<br>The selection of studies from electronic databases was conducted in two stages: at first decision was made based on titles and, where available, abstracts. For studies that appear to meet the inclusion criteria, or in cases when a definite decision cannot be made based on the title and/or abstract alone, the full paper was obtained for detailed assessment against the inclusion criteria. Study quality was assessed by two independent reviewers. If there	9	State the process for selecting studies (i.e., screening, eligibility, included in systematic review, and, if applicable, included in the meta-analysis).	5

was a discrepancy in the decision process the paper was given to the third reviewer to come to consensus.			
**Data collection process:** A standardized data collection form was used to extract title of the study, first author's last name, country where the study was conducted, study design, year of recruitment and follow up, year of publication, sample size, study population, diagnosis and identification of treatment modification, average duration of follow up (for cohort study), potential confounders that were adjusted for, main findings and quality assessment tools. Any data discrepancy was resolved by referring back to the original study.	10	Describe method of data extraction from reports (e.g., piloted forms, independently, in duplicate) and any processes for obtaining and confirming data from investigators.	6
**Data items:** Combinations of key words: ((((((((((((((HIV) OR AIDS*) AND antiretroviral*) OR HAART*) OR ART*) OR ARV*) AND NNRTI*) AND outcomes*) OR treatment failure) OR switch) OR substitution) OR Discontinuation) AND Africa) OR sub-Sahara Africa. The authors were contacted and requested full articles by email when the article was not accessed from these sources.	11	List and define all variables for which data were sought (e.g., PICOS, funding sources) and any assumptions and simplifications made.	4
**Risk of bias in individual studies** Quality assessment of the included studies was also independently performed using the Joanna Briggs Institute Meta-Analysis of Statistics Assessment and Review Instrument (JBI-MAStARI) and Newcastle-Ottawa quality assessment scale by two independent reviewers. The first assessment tool consisted of nine questions. The risk of bias in individual studies was not done.	12	Describe methods used for assessing risk of bias of individual studies (including specification of whether this was done at the study or outcome level), and how this information is to be used in any data synthesis.	5
**Summary measures** Treatment failure defined as either virologic, clinical or immunological failure	13	State the principal summary measures (e.g., risk ratio, difference in means).	6

as per the definition of WHO ART guideline. In addition, studies which used composite outcome as their event also defined as treatment failure. NNRTI substitution was defined as either NNRTI modification, regimen change, NNRTI resistance, or NNRTI discontinuation.			
**Synthesis of results**<br>Heterogeneity among studies was examined using I-squared statistic. According to the test, I-square estimate greater than 50% was considered indicative of moderate to high levels of heterogeneit. Adjusted point estimates were extracted from individual studies and combined together to calculate the pooled estimates. The DerSimonian-Laird random effects method was used to incorporate an additional between-study component to the estimate of variability. If significant heterogeneity was found, and where feasible, subgroup analyses were done to explore differences in outcomes according to study outcomes. The qualitative and quantitative methods were used to present the data extracted from each study. Funnel plot and Egger's test were used to check the presence of publication bias. We plotted the effect by the inverse of its standard error. The symmetry of such plots was assessed both visually, and formally with Egger's test to see if the effect decreased with increasing sample size. Since graphical evaluation can be subjective, we conducted a regression asymmetry test as formal statistical tests for the presence of publication bias.	14	Describe the methods of handling data and combining results of studies, if done, including measures of consistency (e.g., I^2) for each meta-analysis.	7

Section/topic	#	Checklist item	Reported on page #
Risk of bias across studies<br>Funnel plot and Egger's test were used to check the presence of publication bias. We plotted the effect by the inverse of its standard error. The symmetry of such plots was	15	Specify any assessment of risk of bias that may affect the cumulative evidence (e.g., publication bias, selective reporting within studies).	7

assessed both visually, and formally with Egger's test to see if the effect decreased with increasing sample size.			
Additional analyses<br>Subgroup analyses were done to explore differences in outcomes according to study outcomes.	16	Describe methods of additional analyses (e.g., sensitivity or subgroup analyses, meta-regression), if done, indicating which were pre-specified.	7
**RESULTS**			
Study selection<br>A total of 6,394 articles were identified with English-language and human domain restrictions, of which 5,779 were rejected by looking only at the title of the research. The remaining 615 articles were further screened and subsequently, 395 were considered irrelevant or duplicates. The abstracts of 238 articles were then evaluated independently. Of these, 158 records were excluded because of no comparison groups of the outcomes of interest, missing comparison of EFV versus NVP drugs and reviews and meta-analysis	17	Give numbers of studies screened, assessed for eligibility, and included in the review, with reasons for exclusions at each stage, ideally with a flow diagram.	8
Study characteristics<br>All the 16 studies were conducted between 2007 and 2016. Sample size ranges from 167(43) to 27,350 (44) patients. The total number of patients included in all the studies were 70,537, of whom 45,010 (63.8%) were females. The proportion of females ranges from 51% to 72%. Most of the patients, 42,039 (59.6%) initiated with EFV containing regimen. Overall, more females were initiated with NVP containing regimens. The median follow up time was 4 years (IQR: 3-7). Study (45) has the longest follow up time whereas study (46, 47) have followed for shorter period. Almost half of the studies were from South Africa (43-49), the rest were from Kenya (50, 51), Ghana (10, 52), Nigeria (42), Zambia (50), Ethiopia (26, 53) and sub-Sharan Africa (54, 55). Study (50) was a multicenter study (in Kenya, Zambia and Thailand) and data from Kenya and Zambia were taken due to inclusion criteria. A total of 509 and 152 patients were included in Zambia and Kenya respectively. With regard to the study design, most were retrospective cohort (9). Only	18	For each study, present characteristics for which data were extracted (e.g., study size, PICOS, follow-up period) and provide the citations.	9-10

study (43) did not report about age. The minimum and maximum median age for the included studies were 32 (IQR: 28-36) and 40 (IQR: 35-47) years respectively. In almost all studies, high median age corresponds to EFV containing regimen at initiation. The median CD4 cell counts ranges from 67 (IQR: 21-161) to 192 (IQR: 112-324). The median CD4 cell count was smaller for patients who initiated with EFV containing regimen. This might be due to the occurrence of different opportunistic infection among this group of patients and EFV containing regimen had no organ damage like hepatotoxicity and preferred for this group at large to maintain adherence (8). Two studies (26, 43) did not report the median CD4 cell count at initiation. Only two studies (45, 55) reported the log transformed median viral load. NRTI backbones used differed between studies. Stavudine (d4T)/3TC were used in 13 studies and 3 studies did not use this NRTI backbone at all. AZT/3TC was used in 14 studies and 2 studies did not use this backbone at all. TDF/3TC was used less frequently, in only 7 studies. Most (11/16) of the studies used Cox proportional hazards model for the analysis and reported adjusted hazard ratio. Another two studies used stratified and random effect Cox-proportional models. About 7 studies used second model (Conditional logistic regression, Poisson regression, mixed effect model and marginal structural models). Two of the studies further used sensitivity analysis. In general, with the statistical model used, most of the articles utilized appropriate analysis methods			
Risk of bias within studies	19	Present data on risk of bias of each study and, if available, any outcome level assessment (see item 12).	
Results of individual studies<br>Heterogeneity among the studies within the subgroup was tested using I-squared statistics. The I-squared value for treatment failure subgroup was found to be 81.0% (p-value<0.0001) which indicated the presence of heterogeneity between studies. The weight of the studies was reported from random effect model which ranged from 0.31% to a maximum of	20	For all outcomes considered (benefits or harms), present, for each study: (a) simple summary data for each intervention group (b) effect estimates and confidence intervals, ideally with a forest plot.	12

28.28%. The pooled estimate of risk ratio from random effect model was 0.85 (RR=0.85; 95%CI: 0.75-0.88) for EFV than NVP for treatment failure. For NNRTI substitution subgroup, almost all the studies were individually significant except study (43). The I-squared value is 98.9% (p-value=0.0001) which indicates as there is high heterogeneity between studies. The weight of the studies ranges from 0.37% to 38.09%. The pooled estimate from random effect model was 0.57 (RR=0.57; 95%CI: 0.37-0.89) which is consistent with the estimate from fixed effect model

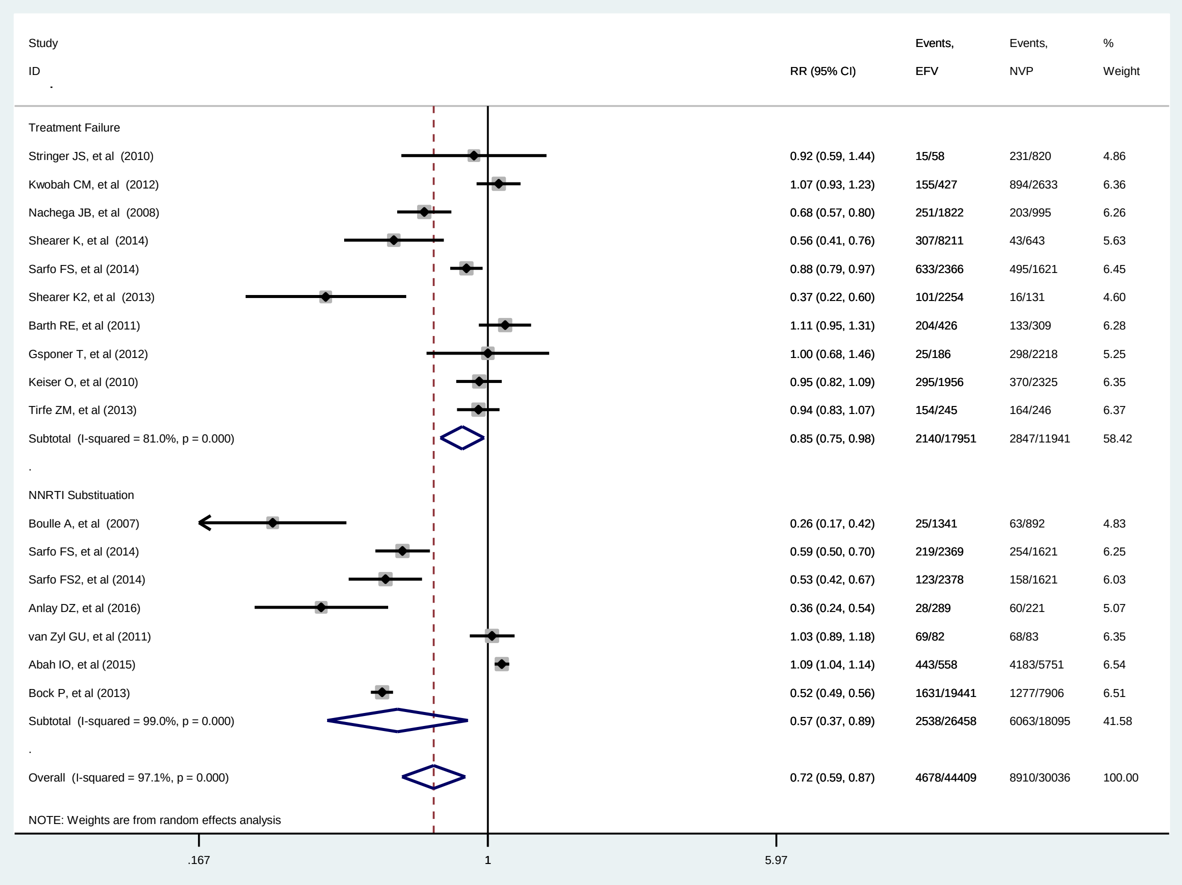

Synthesis of results	21	Present results of each meta-analysis done, including confidence intervals and measures	12

		of consistency.	
Risk of bias across studies<br>One of the main problem in systematic review and meta-analysis is that not all studies carried out are published. Those which are published may be different from those which are not. Research with statistically significant results is more likely to be submitted and published than work with null or non-significant results. This will introduce bias during systematic review and meta-analysis. The presence of publication bias was assessed by funnel plots and tested using Eggers test which is proposed by Egger et al (34) to test for asymmetry of the funnel plot.	22	Present results of any assessment of risk of bias across studies (see Item 15).	9
Additional analysis<br>No additional analysis	23	Give results of additional analyses, if done (e.g., sensitivity or subgroup analyses, meta-regression [see Item 16]).	-
**DISCUSSION**			
Summary of evidence<br>The findings revealed that initiation of ART with EFV containing regimen is associated with a reduced risk of treatment failure (RR=0.85, 95%CI: 0.75-0.98) as compared to nevirapine containing regimen in resource limited settings. The risk ratio of NNRTI switch reduced by 0.57 (95% CI: 0.37-0.89) times for patients who initiated with EFV than NVP.	24	Summarize the main findings including the strength of evidence for each main outcome; consider their relevance to key groups (e.g., healthcare providers, users, and policy makers).	13
Limitations<br>These results need to be interpreted with caution due to limitations. Although a lot of efforts has been made to find more studies, still there were few studies which satisfied the inclusion criteria. The analysis was limited to only articles published in English language; the evidence may not be sufficiently robust to determine the comparative effectiveness of Efaverenz and Nevirapine due to the size of included studies. In addition, the analysis included articles with different definitions of treatment failure and different lengths of follow-up. The reviewed articles have also differences in study design, the type of statistical	25	Discuss limitations at study and outcome level (e.g., risk of bias), and at review-level (e.g., incomplete retrieval of identified research, reporting bias).	14

methods, and the variables included in the analysis. These variations may have resulted in selection bias or low statistical power, thus hindering results.			
Conclusions ‘<br>In conclusion, the finding of this review showed that initiation of ART with EFV containing regimen has reduce risk of treatment failure as compared to NVP containing regimen. In addition, the patients who initiated with EFV are less like to switch than NVP. In contrast, there was about 50% increased risk of death in patients who initiated with EFV as compared to NVP containing regimens. Even though EFV is more expensive to afford for resource poor settings, initiating the patient with EFV containing regimen could be supreme important.	26	Provide a general interpretation of the results in the context of other evidence, and implications for future research.	14
**FUNDING**			
Funding<br>No fund received for this review.	27	Describe sources of funding for the systematic review and other support (e.g., supply of data); role of funders for the systematic review.	None

From: Moher D, Liberati A, Tetzlaff J, Altman DG, The PRISMA Group (2009). Preferred Reporting Items for Systematic Reviews and Meta-Analyses: The PRISMA Statement. PLoS Med 6(7): e1000097. doi:10.1371/journal.pmed1000097

For more information, visit: **www.prisma-statement.org**.
