# Supplementary material for: Choice of initial antiretroviral drugs and treatment outcomes among HIV-infected patients in sub-Saharan Africa: systematic review and meta-analysis of observational studies
Source: Syst Rev. 2017 Aug 25;6:173. doi: 10.1186/s13643-017-0567-7 (PMC5574138; doi:10.1186/s13643-017-0567-7)
Supplement: Supplementary file 2 — Bubble plot with fitted meta-regression line depicting the relationship between the risk of composite outcomes and baseline covariates (proportion of female, baseline CD4 cell counts and follow up period). Fig. S1. Bubble plots of the fitted meta-regression with proportion of female, baseline CD4 counts and follow-up time. (PDF 177 kb) [file 13643_2017_567_MOESM2_ESM.pdf]

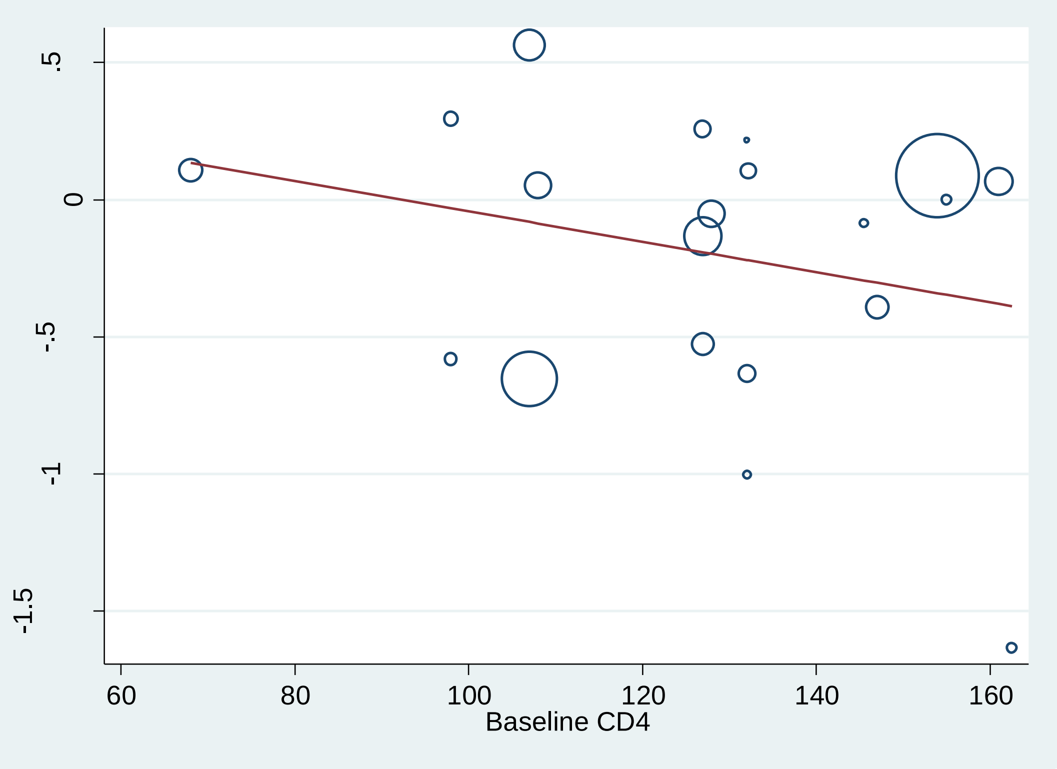

Figure S1. Bubble plot of the fitted meta-regression for baseline CD4 cell counts

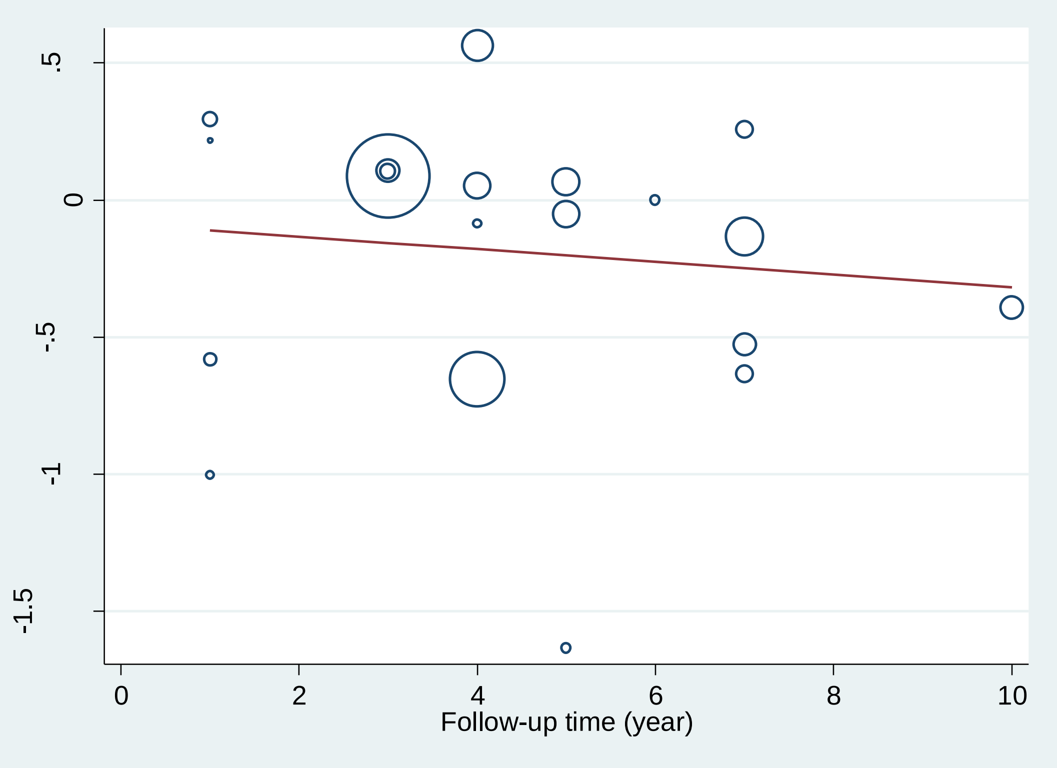

Figure S2. Bubble plot of the fitted meta-regression for follow-up time in years

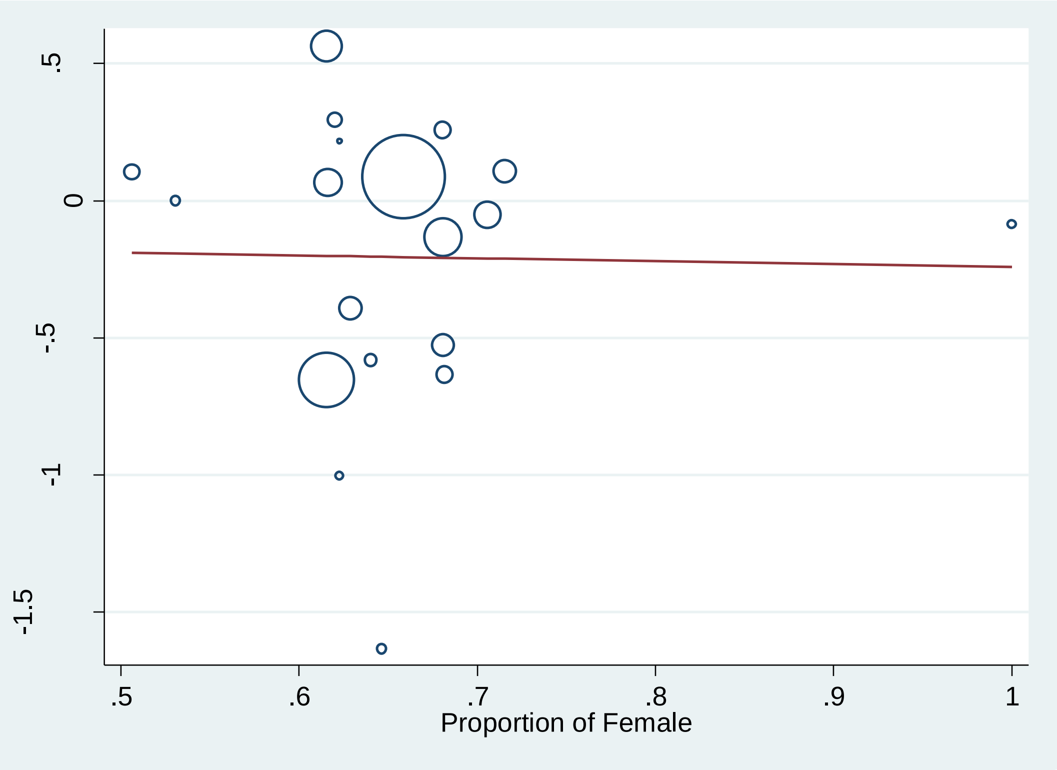

Figure S3. Bubble plot of the fitted meta-regression for proportion of female
